# Efficacy of pure beta tricalcium phosphate graft in dentoalveolar surgery: a retrospective evaluation based on serial radiographic images

**DOI:** 10.1186/s40902-023-00390-w

**Published:** 2023-07-27

**Authors:** Young-Jin Choi, Hoon-Je Chang, Min Jae Kim, Jee-Ho Lee, Bu-Kyu Lee

**Affiliations:** Department of Oral and Maxillofacial Surgery, College of Medicine, Asan Medical Center, University of Ulsan, 88, Olympic-Ro 43-Gil, Songpa-Gu, Seoul, South Korea

**Keywords:** Odontogenic cyst, Socket preservation, Sinus bone graft, Bone graft, Beta-tricalcium phosphate

## Abstract

**Background:**

The use of beta-tricalcium phosphate (beta-TCP) in dental surgery is limited owing to its rapid absorption compared to mixed formulations of hydroxyapatite. However, newly developed pure beta-TCP crystals have demonstrated slow absorption; hence, they last longer within the defect and act as a scaffold until new bone formation. The oral environment is unique and can prove unfavorable for bone grafts due to the high infection rate in the oral cavity and the fragile condition of the oral mucosa. The aim of this study was to evaluate the feasibility of using pure beta-TCP bone grafts in various dental treatments.

**Methods:**

Panoramic X-ray images of 25 patients who underwent bone grafting during dental surgery were analyzed. A specially treated pure beta-TCP crystal, Neo Bone® (Neo Bone®, SN Biologics Co., Ltd, Seoul, Korea), was used in this study. The bone density at the graft site was compared with that of the surrounding bone using the ImageJ software (Wayne Rasband, NIH USA).

**Results:**

Six months after surgery, the bone graft density was similar to that of the surrounding bone in 20 patients and increased in 5 patients. No adverse effects, such as infection, dehiscence, or graft failure, were observed.

**Conclusion:**

The newly developed pure beta-TCP crystal was slowly absorbed and served as support until new bone formation at the defect site, thus demonstrating its potential for use in various oral conditions requiring bone grafting.

## Background

Bone graft materials, such as autografts, allografts, and synthetic substitutes, are widely used in dental surgery to promote bone healing and new bone formation. Although autografts have been used in most cases [1, 2], there is an increasing demand for other bone graft sources due to donor site morbidity and grafted bone volume limitations [2–5]. Xenografts and allografts can be used as alternatives for autografts, but these materials have been associated with cross-infection, low bone regeneration capacity (compared to autografts), and high costs. The use of synthetic materials has gained popularity, mainly because it eliminates the possibility of disease transmission from the donor [6].

Several synthetic materials, including polymers and ceramics, have been proposed for bone grafting. Among them, calcium phosphate ceramics, particularly hydroxyapatite (HA), beta-tricalcium phosphate (beta-TCP), and their combinations, are most commonly used [7]. HA is non-degradable and can maintain the volume at the graft site; however, HA grafts cannot be entirely replaced by newly regenerated bone, which is a drawback in some cases, such as dental implant placement at the graft site. On the other hand, beta-TCP is osteoinductive and can provide osteoconduction for bone formation at the graft site. Furthermore, beta-TCP has excellent biocompatibility and biodegradability, thus making it one of the most potent bone graft substitutes. Additionally, beta-TCP can be resorbed by osteoclasts [7], but it is highly brittle and resorbs quickly owing to its interconnected porous structure [8].

Therefore, pure beta-TCP grafts are required to develop a support system that maintains the mechanical strength during bone union while the grafted bone is resorbed and biodegraded at the same rate as that of the newly formed bone.

The requirements for an ideal scaffold include biocompatibility, biodegradability, mechanical strength, adequate porosity, and sufficient pore dimensions. Beta-TCP has good biocompatibility and biodegradability but low mechanical strength due to its porous structure [9].

**Fig. 1:**
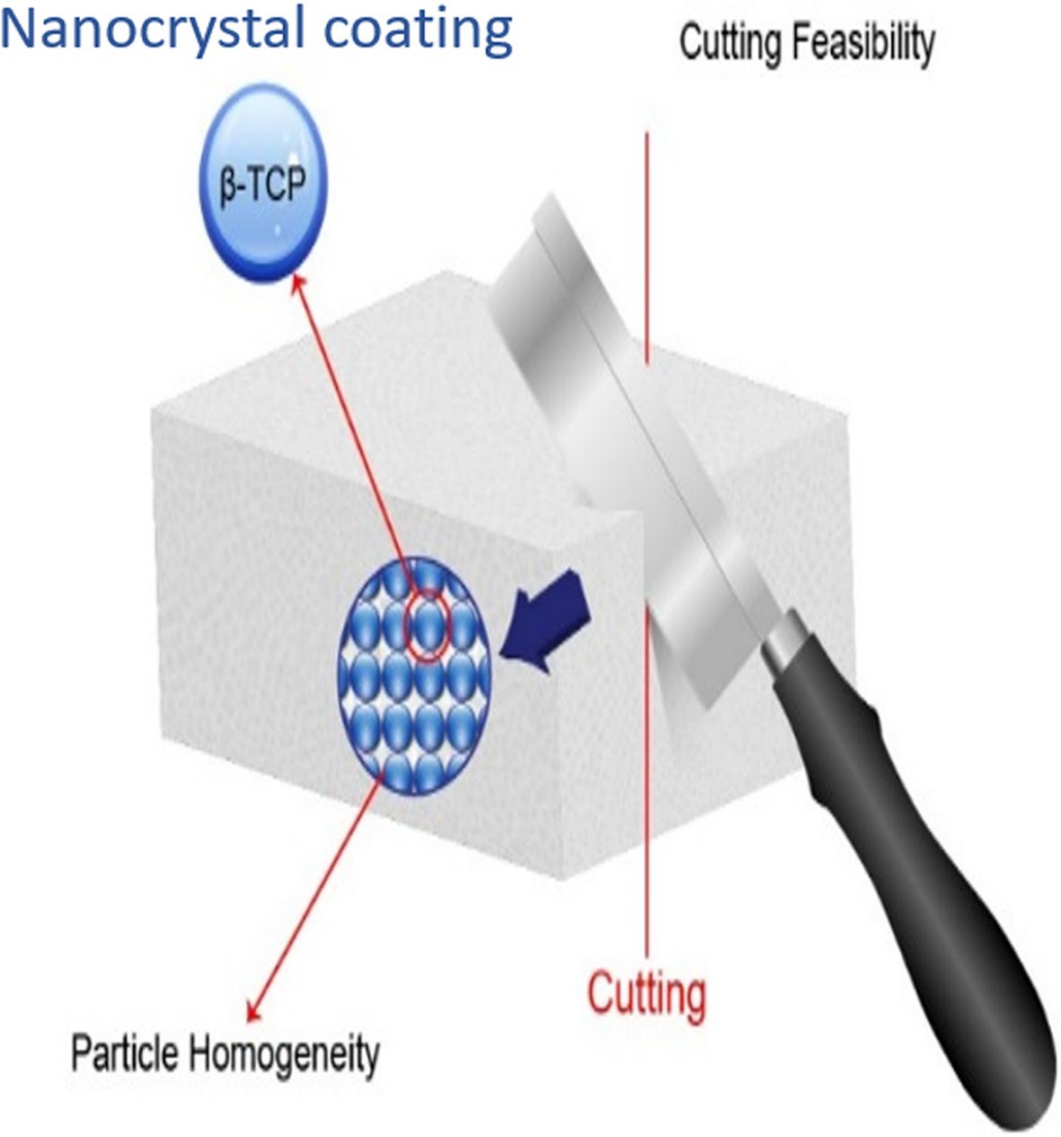
Particle homogeneity can be obtained using nanocrystal coating technology

**Fig. 2:**
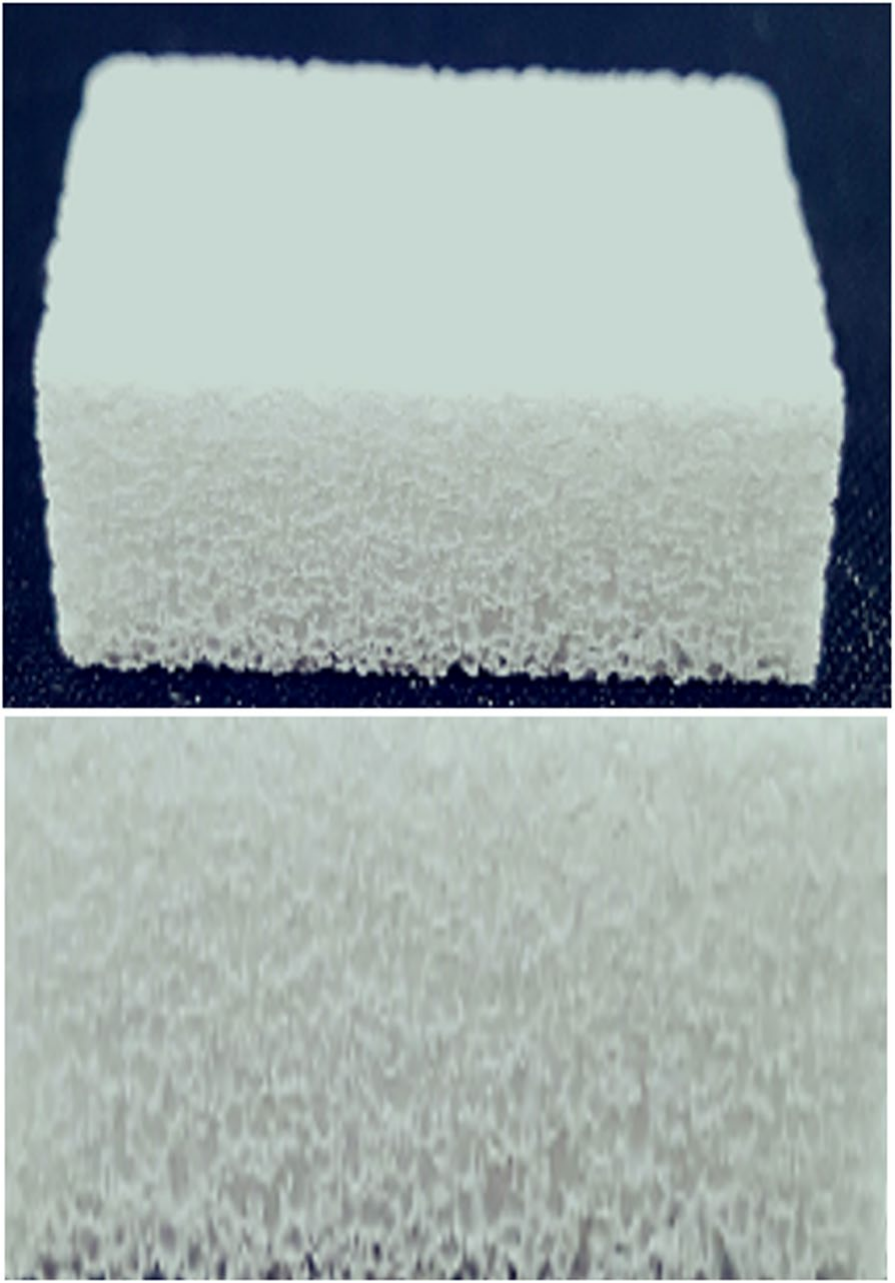
Structure of Neobone. It has a similar structure to the human cancellous bone and improves blood supply

A specially treated pure beta-TCP crystal, Neo Bone® (Neo Bone®, SN Biologics Co., Ltd, Seoul, Korea), was used in this study. Unlike other pure beta-TCP products, Neo Bone® beta-TCP obtains independent structure support through nanocrystal coating. Consequently, the mechanical limitations of existing pure beta-TCP were overcome, and the structure was used to facilitate molding in this study (Fig. 1). Nanoparticle treatment maximizes blood supply by improving the surface area and reproducing the structure of cancellous bone; the absorption rate, structure, and mechanical properties are similar to that of human bone (Fig. 2).

Bone grafting for dental treatments is usually performed using the intraoral approach by elevating the oral mucosa. However, the condition of the oral cavity is not favorable for grafting due to the presence of large numbers of pathologic bacteria and the mechanical forces from food chewing. Moreover, the oral mucosa on the graft site is generally thin and fragile. In addition, the biocompatibility of the pure beta-TCP with the oral mucosal tissues remains unclear. Therefore, the grafts in the oral cavity might be more vulnerable to infection than those used for orthopedic treatment.

This study aimed to evaluate the feasibility of using a pure beta-TCP product for intraoral bone grafting in various dental treatments.

## Methods

The study protocol was reviewed and approved by the institutional review board of the Asan Medical Center, Seoul, Korea (IRB approval No. S2021-2426–0001).

The density of the bone graft material was evaluated and analyzed in 25 bone graft patients after surgery using the pure beta-TCP crystal, Neo Bone® (SN Biologics Co.). The “relative bone density” measurement was used to evaluate the healing process after using the bone graft material, wherein

$$\mathrm{Relative}\;\mathrm{bone}\;\mathrm{density}=\frac{\mathrm{Mean}\;\mathrm{gray}\;\mathrm{value}\;\mathrm{of}\;\mathrm{the}\;\mathrm{defect}\;\mathrm{region}}{\mathrm{Mean}\;\mathrm{gray}\;\mathrm{value}\;\mathrm{of}\;\mathrm{the}\;\mathrm{surrounding}\;\mathrm{bone}}$$

Two panoramic X-ray imaging equipment (Promax, Planmeca, Finland, and CS 8100, Carestream Dental, USA) were used to evaluate the bone grafting sites. The regions of interest (ROI) were determined by free hand selection using the ImageJ software (Wayne Rasband, NIH USA) [10].

### Measurement of bone density and area

The relative bone density was determined by calculating the bone density of the ROI relative to that of the normal bone tissue around the graft. The freehand selection measurement method has a high possibility of error depending on the measurer; therefore, the standard operating procedure method proposed by Manuel was used in this study [10]. This method is free of cost, simple, and easy to compare radiography taken with dental clinic.

One investigator evaluated the repeatability of the measurement by taking re-measurements 2 weeks after the first measurement. The relative bone density was calculated using panoramic radiographs immediately and 6 months after surgery. The value for each sinus was calculated in the case of a bilateral maxillary sinus.

**Fig. 3:**
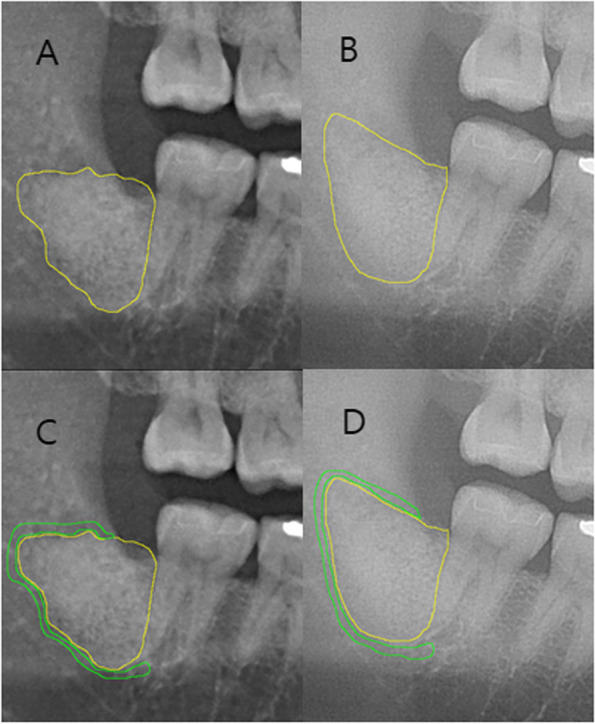
Method for measuring bone density using panoramic radiographs. **A** Postoperation 1st visit defect region. **B** Postoperation 2nd visit defect region. **C** Postoperation 1st visit surrounding bone. **D** Postoperation 2nd visit surrounding bone

As shown in Fig. 3, the densities of the bone graft site and the bone around the site were calculated by marking them on the panoramic radiograph during the 1st visit after surgery. (The area and mean gray values of Fig. 3 are shown in Table 1.) A membrane was applied to prevent the loss of bone graft material, and the graft material was maintained in all the patients.

**Table 1:** Results of Fig. 3

	Area	Mean	Min	Max
A	19,415	132.019	72	167
C	5236	106.779	70	145
B	21,593	163.068	116	189
D	6054	146.206	115	174

### Management of patients

To prevent infection and complications after surgery, the primary closure was performed in all cases. In addition, the antibiotics commonly used after oral surgery, amoxicillin and clavulanic acid, were used three times a day for a week.

## Results

Table 2 shows the relative bone densities calculated immediately (1st visit) and 6 months after surgery. The average grafted area in the 25 patients is shown in Table 3. As shown in Table 4, 11 patients received bone grafts after odontogenic cyst enucleation, 7 received them for socket preservation, 4 for sinus augmentation, and 3 for implants. Eleven cases were in the maxilla, and 7 were in the mandible (Table 5).

**Table 2:** The average relative bone densities in the 25 patients in this study

Patient number	1st visit post-surgery	6 months after surgery
Case 1	1.071	1.182
Case 2	0.839	1.093
Case 3	1.27	1.118
Case 4	0.89	1.039
Case 5	1.119	1.06
Case 6	1.06	0.999
Case 7—right	1.26	1.065
Case 7—left	1.182	1.048
Case 8	1.061	1.017
Case 9—right	1.595	1.531
Case 9—left	1.417	1.225
Case 10	1.087	1.067
Case 11	1.019	1.016
Case 12	1.149	1.274
Case 13	1.592	1.337
Case 14	0.893	0.935
Case 15	1.066	1.098
Case 16	0.914	1.008
Case 17	0.997	1.033
Case 18	1.03	1.083
Case 19	1.227	1.07
Case 20	1.298	1.232
Case 21	1.002	1.001
Case 22	1.069	1.062
Case 23	1.429	1.057
Case 24	1.231	1.004
Case 25	1.214	1.13
Average	1.147 ± 0.2	1.103 ± 0.13

**Table 3:** Average bone graft area in the 25 patients

Patient number	1st visit	6 months later	%
Case 1	23,360	15,366	65.78
Case 2	10,236	11,886	116.12
Case 3	17,868	21,253	118.94
Case 4	15,501	21,253	137.11
Case 5	22,386	21,386	95.53
Case 6	24,972	17,485	70.02
Case 7—right	15,052	14,051	93.35
Case 7—left	16,125	17,206	106.7
Case 8	5823	5618	96.48
Case 9—right	14,677	18,617	126.84
Case 9—left	13,370	12,157	90.93
Case 10	10,740	11,773	109.62
Case 11	9514	8772	92.2
Case 12	26,805	18,328	68.38
Case 13	4041	6953	172.06
Case 14	3123	4158	133.14
Case 15	6035	5345	88.57
Case 16	1841	1041	56.55
Case 17	4837	5058	104.57
Case 18	2617	2408	92.01
case 19	10,154	13,251	130.5
case 20	18,650	19,660	105.42
case 21	14,380	11,981	83.32
case 22	14,153	17,988	127.1
case 23	8914	10,835	121.55
case 24	12,074	10,698	88.6
case 25	14,290	8464	59.23
Average	12,650	12,333	97.5 ± 26.8

The % was calculated by dividing the value immediately after surgery by the value 6 months later × 100

**Table 4:** Dental surgical procedures where the bone graft was used

Cases	Number of patients
Cyst enucleation	11
Socket preservation	7
Sinus bone graft	4
Implant with bone graft	3

**Table 5:** Surgical sites

Surgical sites	Cases
Maxilla	12
Mandible	13

Twenty patients presented with relative bone density values close to 1, whereas the relative bone densities of 5 patients were further away from 1 (Table 6). In addition, the relative densities of the bone graft materials were increased in 9 and decreased in 16 patients at the 6 months after surgery. The area of the graft site was increased in 13 sites and decreased in 14 sites (Tables 2, 3, 4, 5, and 6).

**Table 6:** Changes in bone density

Changes in bone density	Number of patients
Similar	20
Increased	5

No complications or side effects were observed in any of the patients.

## Discussion

Allogeneic, xenogeneic, and synthetic bone have been widely used in dentistry. Among them, synthetic bone has a lower risk of cross-infection and is more competitively priced than the others. However, the clinical use of synthetic bone graft materials is limited because of their structural properties and decreased osteogenic activities [7].

A new synthetic material, beta-TCP, has recently gained popularity owing to its high biocompatibility [11] and osteogenic potential [12, 13]. Nonetheless, it has poor mechanical strength and is easily absorbed [14]; hence, it is usually applied as a mixture with HA to improve the mechanical properties while maintaining the graft volume. HA cannot be replaced with natural bone at the graft site; therefore, a new technology was applied to develop a pure beta-TCP with improved mechanical strength and a lower resorption rate [15, 16].

Neo Bone® is manufactured using the nanocrystal particle treatment method [16], which increases the mechanical strength of the pure beta-TCP by forming uniform particles [17]. Neo Bone® can preserve the high osteogenic potential (osteoconduction and osteoblast activation) of beta-TCP. It ensures high chemical purity and uniformity of the chemical composition and crystal phase by calcifying beta-TCP below the phase transition temperature to minimize the aggregation of raw materials. The micro-crystal coating between the beta-TCP particles guarantees uniformity and improves the mechanical strength and brittleness, so it is not easily broken [17].

Beta-TCP is absorbed by macrophages and multinucleated giant cells [18, 19]. In one study, the volume retention rate of NeoBone® was higher than those of an allogeneic bone graft (survival rate of 80.3% at 6 months after surgery) and a 1:1 mixture of allogeneic and xenogeneic bone (84.1%), thus acting as a scaffold [20]. In another study using conventional pure beta-TCPs, 75.6% of the original graft area was retained 6 months after surgery [20, 21]. Interestingly, 97.5% ± 26.8 of the original graft area remained 6 months after surgery in the current study. This finding indicates that Neo Bone® can achieve preservation of the volume by overcoming the disadvantages of conventional pure beta-TCP grafts. NeoBone® showed a higher volume retention rate 6 months after surgery than the hydroxyapatite and beta-TCP mixture, which retained 82% of the original graft area [17]. Thus, Neo Bone® has served as a support for an extended period until new bone was formed, while the implanted bone was resorbed during the osteosynthesis period. Furthermore, it overcame the clinical weaknesses of existing pure beta-TCP grafts, maintained the volume and height of the bone graft site after surgery, and achieved better volume preservation compared to other bone graft materials.

Conventionally, a mixture of hydroxyapatite and beta-TCP has been used to maintain the volume of the implantation site and improve the mechanical properties [11, 18, 19]. However, conventional synthetic bone to which HA is added has a disadvantage in that the implant must remain in the body for life after bone transplantation. Alternatively, the pure beta-TCP used in the present study not only serves as an adequate scaffold for new bone formation when used alone, but also has the advantage of significantly lowering the possibility of infection caused by the complete absorption of implants at a later point. The findings of the present study indicate that Neo Bone® may be considered as an alternative to a successful bone graft material in various dental surgery cases.

According to the radiological findings, 97.5% ± 26.8% of the Neo Bone® graft area supported volume preservation until 6 months after surgery, and most of the 25 patients had the same or increased relative bone density values. Thus, the graft was gradually replaced by new bone after 6 months.

Histomorphometric analysis to confirm the formation of new bone tissue was not performed for ethical reasons. However, previous studies have reported these findings using histomorphometric analyses of beta-TCP and radiologic evaluations [16, 22].

Okada et al. found that high-purity beta-TCP is safe with excellent osteoconductive properties, even in patients who underwent maxillary sinus augmentation with beta-TCP alone [20] Furthermore, a histological analysis comparing beta-TCP with other materials showed that the rate of new bone formation was significantly increased between 6 months and 1 year, followed by a significant decrease. These findings were corroborated in the present study. Thus, the radiologic data in this study might represent new bone formation in the defect. The relative bone density of the graft was similar to or increased with time when compared to that of the surrounding bone (Table 6). This change in graft density implies that some degree of bone regeneration has progressed into the graft area during the observation period. A retrospective study using a beta-TCP bone graft showed similar results under favorable conditions [23].

Dentoalveolar bone grafting is mainly performed by elevating the oral mucosa in the oral cavity. The oral cavity consists of a large number of bacteria and is exposed to masticatory forces. Furthermore, the mucosa covering the upper part of the graft is mechanically weaker than the skin or muscle layer. These factors increase the likelihood of wound dehiscence at the graft site. Therefore, the infection rate after bone graft during dental treatment is high, and it is expected to be higher in the case of synthetic bone [24]. Accordingly, postoperative systemic antibiotics have been prescribed to reduce infection [25]. No infection or wound dehiscence was observed in the present study; this, Neo Bone® might prove to be biocompatible with the oral mucosa.

**Fig. 4:**
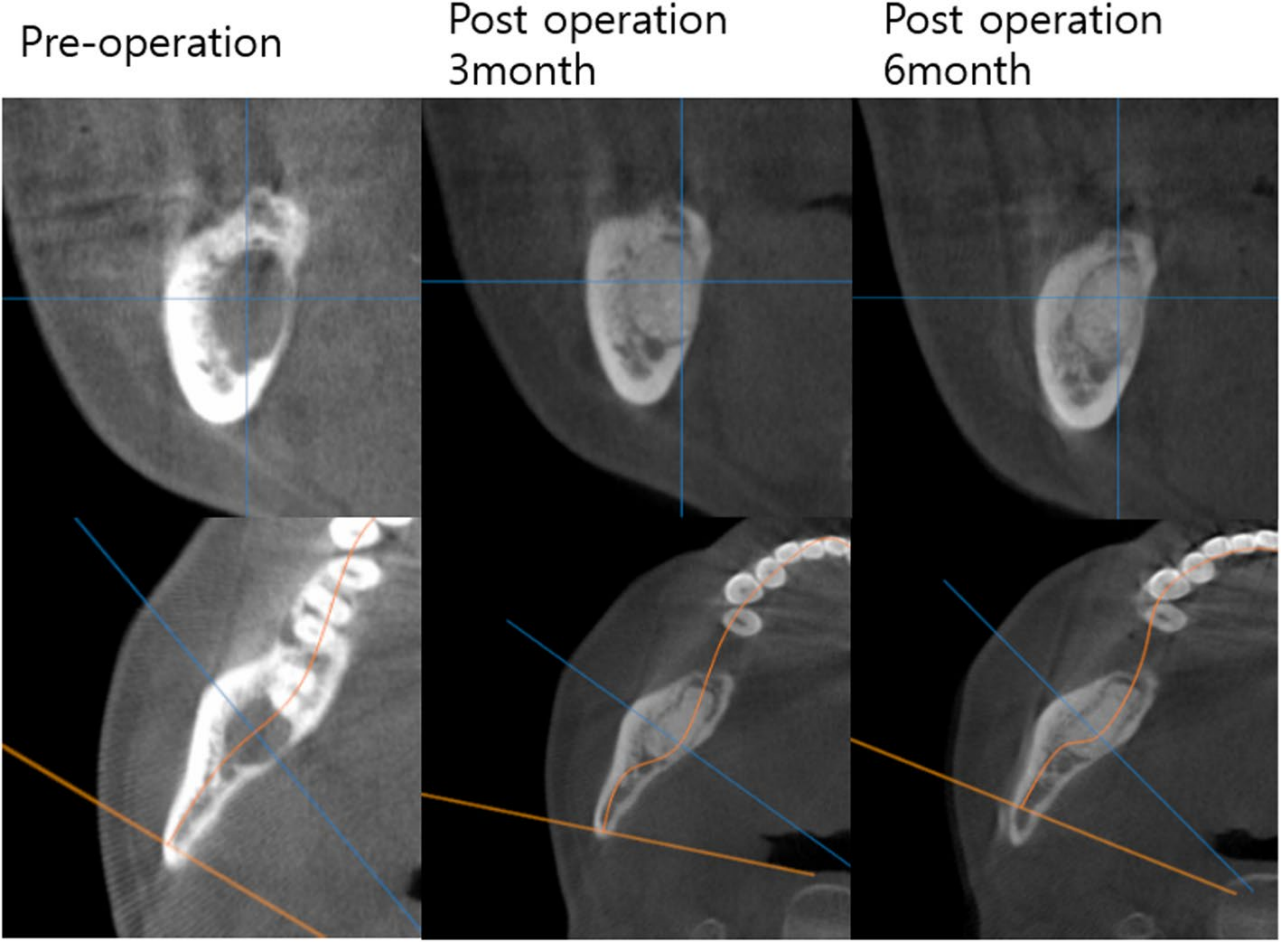
Preoperative and postoperative cone beam CT

The beta-TCP used in this study has the advantage of being able to gradually replace the scaffold with new bone while maintaining its role for a sufficient period of time, unlike conventional beta-TCP. As shown in Fig. 4, it was confirmed that the volume was well maintained up to 6 months after surgery even in CBCT. However, this study has limitations as it did not include histological examination and did not use a control group for retrospective analysis. Therefore, prospective comparative studies with control groups and histological examinations will be necessary in the future.

## Conclusion

In the present study, the specially treated pure-beta TCP, Neo Bone®, could be successfully grafted in various popular clinical dental bone defects. Neo Bone® might be another useful graft option for diverse dentoalveolar bony defects.

## Data Availability

Data sharing is not applicable to this article as no data sets were generated or analyzed during the current study.

## Abbreviations

ROI: Regions of interest
HA: Hydroxyapatite
TCP: Tricalcium phosphate
Beta TCP: Beta tricalcium phosphate
